# Delayed revascularization in acute ischemic stroke patients

**DOI:** 10.3389/fphar.2023.1124263

**Published:** 2023-02-08

**Authors:** T. Marc Eastin, Justin A. Dye, Promod Pillai, Miguel A. Lopez-Gonzalez, Lei Huang, John H. Zhang, Warren W. Boling

**Affiliations:** ^1^ Department of Neurological Surgery, Loma Linda University Medical Center, Loma Linda, CA, United States; ^2^ Department of Pharmacology and Physiology, Loma Linda University, Loma Linda, CA, United States; ^3^ Department of Neurology, Loma Linda University Medical Center, Loma Linda, CA, United States; ^4^ Department of Anesthesiology, Loma Linda University Medical Center, Loma Linda, CA, United States

**Keywords:** ischemic stroke, stroke, revascularization, delayed revascularization, thrombectomy, penumbra, thrombolysis

## Abstract

Stroke shares a significant burden of global mortality and disability. A significant decline in the quality of life is attributed to the so-called post-stroke cognitive impairment including mild to severe cognitive alterations, dementia, and functional disability. Currently, only two clinical interventions including pharmacological and mechanical thrombolysis are advised for successful revascularization of the occluded vessel. However, their therapeutic effect is limited to the acute phase of stroke onset only. This often results in the exclusion of a significant number of patients who are unable to reach within the therapeutic window. Advances in neuroimaging technologies have allowed better assessment of salvageable penumbra and occluded vessel status. Improvement in diagnostic tools and the advent of intravascular interventional devices such as stent retrievers have expanded the potential revascularization window. Clinical studies have demonstrated positive outcomes of delayed revascularization beyond the recommended therapeutic window. This review will discuss the current understanding of ischemic stroke, the latest revascularization doctrine, and evidence from clinical studies regarding effective delayed revascularization in ischemic stroke.

## 1 Introduction

Stroke shares a significant burden of global mortality and disability (Paolucci et al., 2019). According to the Global Burden of Disease (GBD), the estimated lifetime risk of stroke for people aged 25 or above was 24.9% in 2016, with variations reported across different geographical regions (Feigin et al., 2018). A significant decline in the quality of life is attributed to the post-stroke cognitive impairment including mild to severe cognitive alterations (Umarova et al., 2019), dementia, and functional disability, resulting in partial or complete dependence on others (Brainin et al., 2015). An analysis of the GBD (2016) study suggests that stroke accounts for almost 5% of disability-adjusted life years [5]. Socioeconomic damage is a serious repercussion of stroke incidence which is associated with loss of productivity (Simpkins et al., 2020). Therefore, stroke management is vital in preserving neuronal function amid hypoxic damage. In general, the majority of stroke cases are ischemic strokes. Every therapy for ischemic stroke aims at the restoration of blood flow to the endangered brain tissues to effectively reverse hypoxia and salvage the remaining penumbra (Pang et al., 2019). Currently, only two clinical interventions are approved to restore blood flow in ischemic stroke patients including pharmacological and mechanical thrombolysis (Lo, 2008). However, both treatments are limited to the acute phase of ischemic stroke onset with a strict therapeutic window.

Recombinant tissue plasminogen activator (rt-PA) administration is recommended within 4.5 h of stroke onset (Kim et al., 2018). While the endovascular thrombectomy therapeutic window has been extended recently but still remains within a narrow range (Campbell et al., 2019). Time has always been of utmost significance in the management of stroke. The phrase “time is brain” in stroke management refers to the irretrievable loss of nervous tissue with each passing second. In ischemic stroke, the rapid neuronal loss is estimated at 120 million neurons per hour (Saver, 2006). Therefore, time has been the primary focus of every stroke intervention. This gave rise to the “golden hour” in stroke management, a short window of opportunity for effective intervention. The therapeutic intervention of acute ischemic stroke (AIS) were determined based on whether they were presented within the therapeutic window or not (Al Sultan and Hill, 2018). This often resulted in the exclusion of a significant number of patients who could not be treated within such a short time. Efforts have been made to ensure rapid transportation and improve the diagnosis of stroke which has resulted in reduced stroke mortality (Dion, 2004). With improved outcomes, stroke can now be described as a chronic disabling disease. However, still substantial improvement is required to ensure effective stroke interventions in a delayed fashion (Phipps and Cronin, 2020). The purpose of this review is to highlight the pathophysiology of ischemic stroke and present considerations for revascularization with focus on providing evidence of effective delayed revascularization past the acute phase of ischemic stroke.

## 2 Pathophysiology of ischemic stroke

Stroke is defined as focal neurological dysfunction caused by an acute vascular injury of the central nervous system (Pranatasari and Laksono, 2021). The loss of function in stroke is attributed to the interrupted blood supply, resulting in cell death. The prevalence of ischemic stroke is higher which accounts for almost 85% of all stroke incidences (Lakomkin et al., 2019). Embolic arterial occlusion is the primary source of ischemic stroke. Collateral blood flow in the periphery of the occluded vessel can prevent ischemia, however, it is insufficient to maintain neuronal viability (Lakomkin et al., 2019). However, reperfusion of the ischemic region by collateral blood flow can also promote hemorrhagic transformation (Liebeskind, 2003). As hypoxia-induced damage proliferates, it results in irreversible neuronal damage. Several processes are initiated prior to the depletion of oxygen and glucose at ischemic tissue levels (Raichle, 1983). Decreased blood supply initiates a series of alterations at the cellular level, starting with the disruption of ionic homeostasis and culminating in the production of reactive oxygen species (ROS). In ischemia, anaerobic metabolism prevails which reduces the pH, encouraging Na^+^ ions influx to provide a buffering or protective effect (Sanada et al., 2011). As ATP levels deplete, it inactivates ATPase which limits Ca^2+^ efflux, causing the overload of calcium in the cells (Kalogeris et al., 2012).

The critical apoptotic and inflammatory processes start at the sub-acute stage. As a consequence, ROS and cytokine accumulation deteriorate the blood-brain barrier (Sifat et al., 2017). Two types of cell deaths are reported in ischemic stroke, abrupt death or necrosis during the acute phase of stroke onset and programmed cell death or apoptosis over several hours to days (Kanyal, 2015). Foley et al. (2010) reported ischemic transition of brain tissue by voxel analysis regarding penumbra, ischemic core, and infarction volumes. The findings revealed an unchanged penumbra and core during the first 4 h, however, a progressive increase was reported with time. As necrosis is irretrievable damage, apoptosis provides an opportunity to salvage at-risk penumbra from the ischemic core.

Neuroinflammation following an ischemic stroke has emerged both as a friend and a foe. Inflammatory mediators not only exacerbate the injury but also promotes recovery (Jayaraj et al., 2019). Neuroinflammation is secondary to necrosis and apoptosis after the release of immune cells, and microglia. Ischemia activates the brain’s resident microglia which functions as macrophage by phagocytosing injured brain cells (Liu et al., 2017). Microglia produces several pro and anti-inflammatory mediators. For instance, IL-10 produced by microglia and endothelial cells decreases infarct volume, limits cytokines release, and reduces leukocyte adhesion (Lambertsen et al., 2019), whereas HMGB-1 exhibit both beneficial and detrimental effects. Its beneficial effects include endothelial activation and enhancement of neuronal survival whereas damaging effects are manifested as an increase in infarct volume and BBB disruption (Tian et al., 2017).

Inflammation can exacerbate the ischemia-perfusion injury; therefore, inflammation should be monitored during revascularization therapies. Corticosteroids are the preferred approach to suppress inflammatory reactions in vasogenic edema but not in cytotoxic edema as seen in stroke (Emsley et al., 2008). The integrity of neurovascular unit is vital to regulate cerebral blood flow (CBF). Microenvironmental homeostasis of the brain is maintained due to the blood-brain barrier (BBB) which restricts access of blood-borne substances due to tight junctions of endothelial cells (Huang et al., 2020). In ischemic insult, BBB disruption is primarily caused by vessel regression, brain hypoperfusion, and inflammatory responses. BBB degeneration is often manifested as increased paracellular permeability and edema which can further exacerbate the brain injury (Yang et al., 2019).

## 3 Current revascularization practices

Thrombolytic stroke therapy is often linked with the revascularization hypothesis which aims at reopening the occluded vessels to improve regional perfusion in hypoperfused tissues (Rha and Saver, 2007). Restoration of blood to the damaged tissues after an acute ischemic stroke is believed to salvage the tissues without incurring any permanent damage (Pang et al., 2019). From time to time, some challenging questions have also been posed to the revascularization hypothesis. Revascularization of large arteries does not guarantee the expected outcome as distal emboli and microcirculatory occlusion still pose risks. The other concern is the possibility of further damage by revascularization when collateral circulation manages to sustain tissue from serious damage (Rha and Saver, 2007). As the revascularization hypothesis has been endorsed by clinical studies, regulatory agencies have accepted revascularization as treatment option for ischemic stroke to avoid catastrophic outcomes.

For many years, intravenous thrombolytic treatment with tissue plasminogen activator, initiated within 3 h after the presentation of symptoms was the only therapy for acute ischemic stroke (AIS) (Roth, 2011). These recommendations were based on the National Institute of Neurological Disorders and Stroke (NINDS) study in 1995. The NINDS study reported a 30% increase in favorable outcomes for stroke patients who received alteplase within 3 h of the onset of symptoms (Disorders and Group, 1995). Later, two attempts were made to expand the window of treatment up to 6 h by the European Cooperative Acute Stroke Study (ECASS) (Hacke et al., 1995) and ECASS II but they failed (Hacke et al., 1998). Although alteplase resulted in intracranial hemorrhage, the ECASS III study published in 2008 found that intravenous alteplase administration between 3 and 4.5 h was linked with improved clinical outcomes in stroke patients (Hacke et al., 2008). These findings helped expand the thrombolytic time window to 4.5 h from 3 h. However, such a short “golden hour” window presented a huge challenge as most people were excluded from receiving thrombolytic therapy.

In recent times, advances in imaging technologies have allowed better assessment of penumbral imaging and vessel status. Two MRI techniques, perfusion-weighted imaging (PWI) and diffusion-weighted imaging (DWI) are diagnostic measures to select patients with the highest chances of recovery from reperfusion therapy (Neumann-Haefelin et al., 1999). The PWI/DWI mismatch, PWI lesion volume minus DWI lesion volume, are the markers of brain ischemia, showing estimated hypoperfusion (Kakuda et al., 2008). The findings of the DEFUSE study support the PWI/DWI mismatch for identifying candidates for reperfusion therapy. Clinical favorable outcomes were reported in patients who received revascularization based on PWI/DWI mismatch profile compared to those without mismatch (Albers et al., 2006). The advancements in the utilization of imaging techniques and the advent of endovascular techniques have expanded the horizons of AIS revascularization therapy (Touma et al., 2016). Recently, two randomized controlled clinical studies, DAWN and DEFUSE 3 found that endovascular thrombectomy performed up to 16–24 h after the onset of stroke symptoms was effective.

The DAWN trial performed thrombectomy in 107 from a total of 206 participants who had a mismatch between clinical deficit and infarct. The findings reported that at 90 days the utility-weighted modified Rankin scale score was 5.5 in the thrombectomy group as compared with 3.4 in the control group, whereas an improved rate of functional independence of 49% was reported at 90 days by the thrombectomy group compared to 13% in the control group (Nogueira et al., 2017). The DEFUSE 3 study was conducted across 38 U.S. centers, with participants selected based on an initial infarct size of fewer than 70 ml. The mortality percentage was significantly lower in the endovascular therapy group (14%) compared to the medical therapy group (26%), whereas the modified Rankin scale score was also improved in the endovascular therapy group (Albers et al., 2018). This evidence paved the way for an official guideline from the American Heart Association/American Stroke Association in 2018. The updated recommendations extended the window of opportunity to 24 h in select candidates through the utilization of computed tomography perfusion (CTP) and magnetic resonance imaging (MRI) by assessing the infarct core and salvageable penumbra (Powers et al., 2018).

## 4 Clinical evidence on delayed revascularization

The concept of delayed revascularization is based on identifying salvageable tissue during the intervention. The relationship between accurate identification of ischemic penumbra and successful clinical outcome of revascularization has been demonstrated (Toth and Albers, 2009). The rate of loss of salvageable tissue varies between individuals, primarily due to the extent of collateral blood flow. Cerebrovascular neuroimaging can contribute to the identification of select patients who have adequate collateral circulation. Magnetic resonance imaging (MRI) is the predominant tool to diagnose infarct core and ischemic penumbra (González, 2012).

The scope of this review includes potential studies that demonstrated successful revascularization of large vessel occlusion at 24 h or more after the onset of stroke symptoms (Table 1). Some studies indicate that significant salvageable penumbra remains even after 24 h of initial manifestations of symptoms (Baron, 2021). Based on the imaging techniques, some select patients can be candidates for delayed revascularization. A review focused on late revascularization in patients with refractory chronically occluded internal carotid artery (COICA) improvement in clinical outcomes in such patients. Successful revascularization improved blood circulation (perfusion) in salvageable penumbra (Zanaty et al., 2020). The study results are based on 18 studies and 389 patients who underwent endovascular technique (ET) or hybrid surgery (ET plus carotid endarterectomy). The intervention time to the onset of symptoms was 15 days to 28 months. The participants were divided into 4 groups as A, B, C, and D, based on occlusion types including tapered occlusion of ICA (A), abrupt occlusion of ICA (B), completely absent ICA from the bifurcation with supraclinoid reconstitution (C), and completely absent ICA from the bifurcation without supraclinoid reconstitution (D). The highest revascularization rates were shown by types A and B (95.4%). The recanalization rates for Type C and D were 87.6% and 29.8% respectively. The findings had successful revascularization improved cognitive parameters in patients (Zanaty et al., 2020).

**TABLE 1:** Clinical evdences of effective delayed recanalization in ischemic stroke patients.

References	Occlusion location	Duration of symptoms	Participants	Outcome/findings
Vang et al. (1999)	Proximal MCA (59%)	>24 h	21	62% (13) had improved CMCT
	Distal MCA (41%)			
Huded et al. (2015)	BA	26 h	6-year-old boy	mRS: 0 at both 10 days and 3 months
				After 3 months, the boy is independent for ambulation and daily activities.
Cognard et al. (2000)	BA	36 h	8-year-old old child	Three months later, the boy was completely asymptomatic, and an MRA showed a normal appearance of the basilar artery
				Clot angioplasty allows partial revascularization
Ishibashi et al. (2013)	MCA, ACA	25–54 h	4	50% (2) mRS of 0 in MCA; 100% (1) mRS of 0 in ACA
Bodey et al. (2014)	BA (3)	BA cases (17–36 h) left MCA case < 6 h	4 (5–15 year)	The modified rankin score (mRS) for Children was 3.
	Left middle cerebral artery (MCA) (1)			50% had MRS 0.
Condie et al. (2012)	BA	46 h	5-year-old boy	Mild incoordination of right arm.
				Delayed intra-arterial thrombolysis and vertebral artery coiling can be successfully used to treat basilar artery occlusion.
Wilkinson et al. (2018)	BA	50 h	17-month-old child	At 3 month follow-up, the patient had a modified Rankin Scale score of 0.
Grigoriadis et al. (2007)	BA	50 h	6-year-old child	Complete recovery, Rankin scale score: 0
				Intra-arterial thrombolysis combined with cerebral angioplasty, is effective way to restore the patency of the basilar artery.
Janmaat et al. (2009)	VBA	60 h	4-year-old boy	After 1 year, he had minor atxia, the modified Rankin scale score was 1.
Xavier et al. (2012)	ICA	6 days	16-year-old boy	At the 3 month follow-up, his modified Rankin Scale score was 1 and at 1 year it was 0.
Yao et al. (2019)	BA	5 days	32	The median score reduction in mRS from baseline at 90 days was 1.0.
Ishihara et al. (2006)	ICA	30 days	72-year-old male	At 6-month follow-up no cerebral ischemic events were observed, and the ICA was patent.
				Preoperative minimental score was 23/30, and this score improved to 28/30.
Zhang et al. (2019)	ICA	17–120 days	30	Periprocedural complications included recurrent laryngeal nerve injury in one patient and intracranial hemorrhage in one. mRS score of 2.5 ± 0.6 at 3 months. Preprocedural mRs score was 3.4 ± 0.6.
Thomas et al. (2007)	ICA	0.75 months	60-year-old man (1)	Both patients were asymptomatic at 30 days
			70-year-old man (1)	
Bhatt et al. (2009)	ICA	∼32 days	55-year-old woman	At the 9-month follow-up, she remains neurologically intact
Yu et al. (2007)	BA	80 days	55-year-old male	CT angiography 6 months later showed patent BA with excellent distal flow.
Wu et al. (2018)	ICA	3 months	36	Patients with late SR of the ICA appear to have preserved functional ability and favorable clinical outcomes.
				18 months follow-up mRS: 2.
Terada et al. (2005)	ICA	79 days	73-year-old male	Endovascular revascularization by using an embolic protection device can be an alternative treatment for symptomatic ICA occlusion.
				At 4 months follow up no new neurological deficit or TIA.

### 4.1 Delayed revascularization in basilar artery occlusions (BAOs)

Pediatric arterial stroke poses a significant challenge as it is different in terms of etiology, clinical presentation, and management compared to adults. An estimated 1.2–7.9 among 100,000 children undergo a stroke event annually (Fullerton et al., 2003). Childhood basilar artery (BA) occlusion has poorer outcomes, especially in absence of revascularization therapy. A case report of a 6-year boy with recurrent posterior circulation stroke secondary to basilar artery occlusion had successful revascularization at 26 h after worsening of the symptoms. Upon arrival, MRI revealed acute infarction in the left thalamus, with a Pediatric NIHS Score (Ped NIHSS) of 15. The patient’s NIHSS was improved to zero with mRS of 0 on Day 10. After 3 months the boy was independent in daily activities (Huded et al., 2015). Although BA occlusion has a poor prognosis, intra-arterial thrombolysis for revascularization can produce positive clinical outcomes (Cognard et al., 2000). Cognard et al. (2000) reported a case of an 8-year-old child with total BA occlusion. Although an of the basilar artery performed by balloon catheter after 36 h of manifestation of symptoms was only successful in partially recanalizing the BA, perforating arteries of the brain stem were seen on angiography. At 3 months follow up, the child had a complete recovery with normal neurological function. The study advocated that revascularization of BA can be performed even later than 24 h after the onset of symptoms. Even partial revascularization can increase the efficacy of thrombolysis.

Pediatric acute ischemic stroke (AIS) is a significant cause of acute neurological symptoms. Bodey et al. (2014) performed a series of mechanical thrombectomy cases in children in the United Kingdom, 17–36 h after the onset of symptoms. A total of 4 cases (5–15 years) with PedNIHSS > 17 presented with BA occlusion (3) and MCA (1). At 6 months follow up, all children had returned to mainstream schools, with a modified Rankin score (mRS) of 0–3, and 50% had mRS of 0. The studies have shown that pediatric AIS can be done by delayed revascularization in select patients (Bodey et al., 2014). A similar case of recurrent basilar artery occlusion in a 5-year-old boy was treated with emergency intra-arterial tPA. However, upon manifestation of adverse symptoms, mechanical thrombolysis of basilar artery occlusion was performed after 36 h of hospital admission. Delayed intra-arterial thrombolysis alleviated basilar artery occlusion. At the 3-month follow up the boy has only mild incoordination of his right forearm and hand (Condie et al., 2012).

The occlusion of BA in children is a rare occurrence (Voetsch et al., 2004). The leading cause of BA occlusion in children is vertebral artery (VA) dissection. A case of a 17-month child with basilar artery occlusion was reported by Wilkinson et al. (2017), treated with mechanical thrombectomy after 50 h of onset of clinical symptoms. Initial MRI was suggestive of acute cerebellar, occipital, and thalamic infarct, along with left vertebral artery occlusion. At the 3-month follow-up, the patient had a modified Rankin Scale score of 0 (Wilkinson et al., 2017). A case study of locked-in syndrome was presented by Grigoriadis et al. (2007) of a 6-year-old boy with BA occlusion presented 44 h after the onset of symptoms. BA was partially recanalized after 6 h of admission. Prompt recovery was reported after 12 h of the procedure, with fully restored speech function. Intra-arterial thrombolysis combined with cerebral angioplasty found potential to treat ischemic stroke in children up to 50 h after clinical symptoms (Grigoriadis et al., 2007). In adults, BA occlusion has devastating outcomes with a higher fatality rate of 83%–92%, however, clinical success is often associated with site of occlusion (Grigoriadis et al., 2007). Yu et al. (2007) reported a case of a 55-year-old male with BA occlusion. MRI investigation revealed multiple infarcts in the posterior circulation. The site of occlusion was successfully recanalized by thrombolysis and stent placement, 80 days after the onset of symptoms. CT angiography 6 months later resulted with a patent BA with excellent distal flow (Yu et al., 2007).

### 4.2 Delayed revascularization in the internal carotid artery (ICA) occlusion

About 4%–15% of all ischemic stroke is caused by internal carotid artery occlusion (Mayer et al., 2020). Spontaneous revascularization of an acutely occluded internal carotid artery (ICA) is a rarely reported phenomenon as ICA occlusion exhibits a high risk of stroke recurrence (Gorelick et al., 2008). Xavier et al. (2012) treated a case of a 16-year-old boy with internal carotid artery occlusion 6 days after the appearance of symptoms. Revascularization was done *via* mechanical thrombectomy and self-expanding stent. Perfusion of the penumbra was achieved after the revascularization procedure. The mRS score was 1 and 0 at 3 months and 1 year respectively (Xavier et al., 2012). Non-acute atherosclerotic intracranial large artery occlusion (ILAO) is a type of large artery intracranial occlusive disease. Yao et al. (2019) reported 32 consecutive cases of intracranial large artery occlusion (ILAO) after 24 h of manifestations of clinical symptoms. The mean time for all patients from imaging diagnostic to treatment was 25.5 days. The overall success rate for revascularization was 53.1%. The mRS score improve by a value of 1 in recanalized group compared to the failure group. The study found that for select participants, delayed revascularization beyond 24 h is feasible (Yao et al., 2019). Often, revascularization of chronic anterior circulation occlusions is contraindicated due to the fear of exacerbation of damage. However, a case of a 72-year man with intracranial internal carotid artery occlusion was successfully recanalized after 30 days of onset of symptoms. At the 6-month follow-up, no cerebral ischemic events were observed, and the ICA was patent. The preoperative mini-mental score was 23/30, and this score improved to 28/30 (Ishihara et al., 2006).

Zhang et al. (2019) reported delayed revascularization 17–120 days after the onset of clinical symptoms. 65 patients with internal carotid artery occlusion were analyzed in the study. The success of delayed revascularization was 100% in all cases, with significant improvement in mRS score, from mean mRS score of 3.4 to 2.5 (Zhang et al., 2019). Two cases of 60-year-old and 70-year-old males, with internal carotid artery occlusion, were recanalized by Thomas et al. (2007). The mean duration from the onset of symptoms to clinical intervention was 22 days (Thomas et al., 2007). Angioplasty and stent placement were used for revascularization in both cases. Both patients were asymptomatic at 30 days. Often, the treatment of chronic large vessel occlusion is limited to medical management. A 55-year-old woman had a totally occluded right internal carotid artery. She was initially treated medically with antiplatelet therapy. However, upon the manifestation of newer clinical symptoms with failure of medical therapy, she was treated with angioplasty and stent placement, 32 days after the onset of initial symptoms. At the 9-month follow-up, she remained neurologically intact (Bhatt et al., 2009).

Rare cases of late spontaneous revascularization of internal carotid artery (ICA) have been reported. Wu et al. (2018) reported the late spontaneous revascularization (SR) of ICA occlusion in 36 patients. The follow-up of the patients was done for more than 18 months. Three patients (8.3%) had late spontaneous revascularization (SR), which occurred in the 3rd, 16th, and 18th months after the occlusion. Of these patients, one suffered from re-occlusion of the recanalized ICA without presenting with any novel significant symptoms. Patients with late SR of the ICA appear to have preserved functional ability and favorable clinical outcomes. 18 months follow-up mRS score was 2 (Wu et al., 2018). In a case of study by Terada et al. (2005), a 73 old male with ICA occlusion presenting after 79 days post clinical symptoms appeared. The totally occluded ICA was completely recanalized through percutaneous transluminal angioplasty with a stent. Endovascular revascularization by using an embolic protection device can be an alternative treatment for symptomatic ICA occlusion. At 4 months follow up, there was no new neurological deficit or TIA. Ishibashi et al. (2013) attempted delayed revascularization by high-dose argatroban therapy in four patients after more than 24 of the onset of symptoms. Argatroban 30 mg was administered for first 15 min, followed by 90 mg for 60 min and 60 mg for 60 min, respectively. The success rate for delayed revascularization was 100% in the study. The study report that high-dose argatroban deactivates thrombin and provides an anticoagulant effect. However, the inadequate dosage of argatroban can cause hemorrhage. Another study reported the case of a 4-year-old boy with vertebrobasilar artery thrombosis who presented after 60 h of clinical symptoms. Patient had prominent dysarthria and dysphagia. Upon MRI investigation, abnormalities were found in the pons region, further confirmed as vertebrobasilar artery thrombosis. Intra-arterial thrombolysis was performed with urokinase. After 1 year, the boy was able to attend school with minor ataxia (Janmaat et al., 2009).

The brain has an intrinsic self-repair mechanism after a stroke. Some studies demonstrate the potential of the brain to regenerate and reorganize after a localized injury (Lee and van Donkelaar, 1995). The formation of novel pathways by surviving neurons and activation of existing yet inactive pathways seems to be predominant pathophysiological mechanism of brain plasticity (Stein et al., 1997). Similar findings were presented by Vang et al. (1999). The authors presented a case of absent motor response on day 1 but the response reappeared on day 14. They also found delayed revascularization in 38 patients with acute ischemic stroke involving proximal MCA (59%) and distal MCA (41%) occlusion. They found successful revascularization of 38% of patients after 24 h from the onset of symptoms. The central motor conduction time (CMCT) the time taken by neural impulse from CNS to muscles improved in 87% who were recanalized before 24 h compared to 62% after 24 h (Vang et al., 1999).

### 4.3 Delayed revascularization in animal studies

The therapeutic window in ischemic stroke has not been challenged beyond certain critical time limits due to the associated risk of reperfusion injury (Zhu et al., 2020), hemorrhagic transformation risk (Kanazawa et al., 2017), early arterial re-occlusion (Santana et al., 2020), and other deleterious effects on subjects. In recent years, animal models have been widely utilized to improve the understanding of the physiopathology of stroke. Both animal and human studies have demonstrated the protective effects of delayed recanalization to salvage the at-risk penumbra and reduce neurological deficits attributed to ischemic stroke. However, the underlying cellular mechanisms that contribute to improved outcomes after delayed revascularization are poorly understood. As ischemic stroke has emerged as a multi-phasic disorder, several novel investigations have revealed various cellular factors such as macrophage infiltration, neural stem/progenitor cells, fibroblast growth factor 21 (FGF21), and tyrosine kinase receptor (c-Met)/hepatocyte growth factor (HGF) that contribute to angiogenesis and repair of the infarct core after delayed recanalization (Cai et al., 2020). Macrophage infiltration of the ischemic core has been shown to promote neurovascular remodeling in ischemic stroke (Wang et al., 2020). Pang et al. (2022), in a study, showed that delayed recanalization at day 3 postinfarct improved vasculature of the ischemic core while reducing infarct volume and neurological outcomes. Their investigations revealed that macrophage infiltration after recanalization of middle cerebral artery occlusion (rMCAO) is responsible for increased neurovascular remodeling which was confirmed by the increased presence of vascular endothelial growth factor A and platelet-derived growth factor B—markers of pro-angiogenic activity. The investigators further evaluated the responses of macrophage depletion and found significantly reduced levels of angiogenesis, inflammation, and neuronal survival which resulted in adverse outcomes following delayed recanalization (Pang et al., 2022).

Similar findings were shared by Kang et al. (2022) who reported that delayed recanalization at 3 days after rMCAO resulted in increased polarization of M2 microglia in the ischemic core, decreased inflammation, and improved neurological outcomes. The authors exhibited improved microglial polarization through IL-4R (interleukin-4 receptor)/STAT6 (signal transducer and activators of transcription 6)/PPARγ (peroxisome proliferator-activated receptor γ) pathways in rats (Kang et al., 2022). STAT 6 promotes PPARγ pathways in macrophages and has been shown to contribute to inflammation resolution (Lee et al., 2021). McBride et al. (2018), in their study, concluded that delayed recanalization performed at 3, 7, or 14 days after pMCAO in male rats restored blood flow, improved neurological outcomes, reduced infarct volume, and increased neural stem/progenitor cells within the infarction (McBride et al., 2018). Zheng et al. (2019) demonstrated that delayed recanalization at 3 days after permanent middle cerebral artery occlusion (MCAO) decreased infarct volumes and improved neurological outcomes in rats. They also found increased levels of fibroblast growth factor 21 (FGF21) after the recanalization of MCAO. The investigators concluded that FGF21 activated the FGFR1/PI3K/Caspase-3 signaling pathway which attenuated neuronal apoptosis in the penumbra (Zheng et al., 2019). Recently, FGF21 has been shown to reduce cell apoptosis in the liver and heart ischemic injury by activating fibroblast growth factor receptor 1 (FGFR1) and downstream signaling pathways (Cong et al., 2013). Activation of tyrosine kinase receptor (c-Met) by hepatocyte growth factor (HGF) showed an anti-apoptotic effect in numerous disease models by regulating a variety of biological processes such as vascular remodeling, epithelial-to-mesenchymal transition, and cellular motility (Chaparro et al., 2015). Tang et al. (2020) reported that delayed recanalization increased HGF expression, reduced infarct volume, and neuronal apoptosis after MCAO, partly *via* the activation of the HGF/c-Met/STAT3/Bcl-2 signaling pathway (Tang et al., 2020).

## 5 Conclusion and future directions

There is existing evidence in the literature that supports improved clinical outcomes after subacute revascularization in select patients with favorable imaging characteristics. However, several limitations need to be addressed such as sample size, diagnostic precision, and standard protocols. Improvements in diagnostic tools may help identify salvageable penumbra with greater accuracy. Randomized clinical trials should be conducted to investigate the underlying therapeutic mechanisms for delayed interventions which may further extend the time window for revascularization in acute ischemic stroke patients.
